# Dietary Habits Are Associated With School Performance in Adolescents

**DOI:** 10.1097/MD.0000000000003096

**Published:** 2016-03-25

**Authors:** So Young Kim, Songyong Sim, Bumjung Park, Il Gyu Kong, Jin-Hwan Kim, Hyo Geun Choi

**Affiliations:** From the Department of Otorhinolaryngology – Head and Neck Surgery, Seoul National University Hospital (SYK); Department of Otorhinolaryngology – Head and Neck Surgery, CHA Bundang Medical Center, CHA University, Seoul (SYK); Department of Statistics, Hallym University, Chuncheon (SS); Department of Otorhinolaryngologyn – Head and Neck Surgery, Hallym University Sacred Heart Hospital, Anyang (BP, IGK, HGC); and Department of Otorhinolaryngology – Head and Neck Surgery, Hallym University Kangnam Sacred Heart Hospital, Seoul, Republic of Korea (J-HK).

## Abstract

Several studies suggest that dietary habits are associated with poor academic performance. However, few studies have evaluated these relations after adjusting for numerous confounding factors. This study evaluated the frequency of various diet items (fruit, soft drinks, fast foods, instant noodles, confections, vegetables, and milk) and the regularity of meal times (breakfast, lunch, and dinner) all at once.

A total of 359,264 participants aged from 12 to 18 years old were pooled from the Korea Youth Risk Behavior Web-based Survey (KYRBWS) for the 2009 to 2013 period. Dietary habits over the last 7 days were surveyed, including the regularity of consuming breakfast, lunch and dinner and the frequency of eating fruits, soft drinks, fast foods, instant noodles, confections, vegetables, and milk. Physical activity, obesity, region of residence, subjective assessment of health, stress level, economic level, and parental education level were collected from all of the study participants. School performance was classified into 5 levels. The adjusted odds ratios (AORs) of dietary habits for school performance were analyzed using multinomial logistic regression analyses with complex sampling. Structural equation modeling was used to analyze the effects of diet factors on school performance while considering the effects of other variables on both diet factors and school performance.

Frequent intakes of breakfast (AOR = 2.34, 95% confidence interval [CI] = 2.20–2.48), fruits (AOR = 1.73, 95% CI = 1.62–1.86), vegetables (AOR = 1.48, 95% CI = 1.37–1.61), and milk (AOR = 1.35, 95% CI = 1.28–1.43) were related to high levels of school performance (each with *P* < 0.001). In contrast, soft drinks (AOR = 0.42, 95% CI = 0.38–0.46), instant noodles (AOR = 0.62, 95% CI = 0.55–0.70), fast food (AOR = 0.83, 95% CI = 0.72–0.96), and confectionary (AOR = 0.86, 95% CI = 0.80–0.93) were negatively associated with school performance (each with *P* < 0.001).

This study confirms previous studies of school performance and dietary habits that find a positive association with eating breakfast and consuming fruits and milk and a negative relation with soft drinks, instant noodles, fast foods, and confections.

## INTRODUCTION

Various factors, such as physical activity, obesity, stress, and income level, are related to school performance in previous studies.^1,2^ Stress levels and parental factors (parental education levels, aspirations for their children's school performance, and family income) are also related to the school performance of their children.^3,4^ Dietary habits are also associated with school performance. For example, fast foods affect academic performance.^5^ These foods are notorious for their poor nutrient quality, and they often do not meet nutrient guidelines.^6^ Moreover, >50% of fast food meals exceed recommendations for sodium, and <25% of these meals met guidelines for *trans* fats. Less than 1/3 of fast food meals provided adequate calcium and iron, and <20% provided adequate vitamin A.^6^ Insufficient nutrient intakes, particularly of iron, and high intakes fat and added sugar due to frequent fast food meal consumption are known to be associated with poor school performance and metabolic diseases, such as insulin resistance and obesity in other studies of children.^5,7,8^ The frequent skipping of breakfast is another dietary habit that may have detrimental effects on adolescents, and children show enhanced spatial and/or short-term memory after eating breakfast, probably due to the facilitated blood glucose response following a meal.^9^ Several studies emphasize the importance of eating breakfast for cognition and learning due to the gradual release of energy for brain function and intakes of micronutrients, particularly iron, iodine, and vitamin A, irrespective of other covariates, including behavioral factors in children and adolescents.^10,11^ Diet habits are important in adolescents because they have high brain metabolic needs. Brain glucose consumption is higher in children until the age of 16 to 18 years than in adults.^12^ Furthermore, both the nutritional components of each food and the overall quality of diet, as represented by high diet quality index values or healthy dietary habits, are suggested to be related to academic performance.^13^ This is because overall diet quality may reflect one's socioeconomic status (SES) and other personal characteristics that could influence academic performance.

The present study was aimed at analyzing the dietary habits that are related to school performance. We were especially concerned with the frequency of fast food, instant noodle, and confection consumption, as well as the regularity of meal times and patterns such as skipping breakfast, which are common among adolescents. Several demographic and socioeconomic factors, as well as dietary habits, were considered in this study. These adjustment strategies enable us to identify the relations between dietary habits and academic performance more clearly. Moreover, we reciprocally adjusted the dietary factors themselves. Our search revealed no study that simultaneously evaluated the associations among the consumption of fruits, soft drinks, fast foods, instant noodles, confections, vegetables and milk, regular consumption breakfast, lunch, and dinner and academic performance among adolescents. Based on a large, representative population-based dataset, we could obtain reliable results for the associations between each dietary habit and academic performance.

## MATERIALS AND METHODS

### Study Population and Data Collection

The Institutional Review Board of the Centers for Disease Control and Prevention of Korea (KCDC) approved this study (2014-06EXP-02-P-A). Written informed consent was obtained from each participant prior to the survey. As this web based survey was performed at the school with huge participants, the informed consent from their parents was exempted. This consent procedure was approved by the IRB of KCDC.

This study is a cross-sectional study using data from the Korea Youth Risk Behavior Web-based Survey (KYRBWS). This study covers one nation using statistical methods based on designed sampling and adjusted weighted values. The KYRBWS waves conducted in 2009, 2010, 2011, 2012, and 2013 were analyzed. The data were collected by the KCDC. Korean adolescents from 7th through 12th grades completed the self-administered questionnaire voluntarily and anonymously. The validity and reliability of the KYRBWS has been documented by other studies.^14,15^ Using 43 regions (considering the administrative district, geographic accessibility, number of schools, and population size) and schools, the population was stratified into 129 levels for sample distribution and then the sample was selected using stratified, 2-stage (schools and classes) clustered sampling based on Education Ministry data. The teachers of the selected classes registered the number of participants online. Then students were then asked to participate in the survey using school's internet access. Sampling was weighted by statisticians, who performed poststratification and considered both nonresponse rates and extreme values. Detailed methods were described on the KYRBWS website (http://yhs.cdc.go.kr/).

**FIGURE 1:**
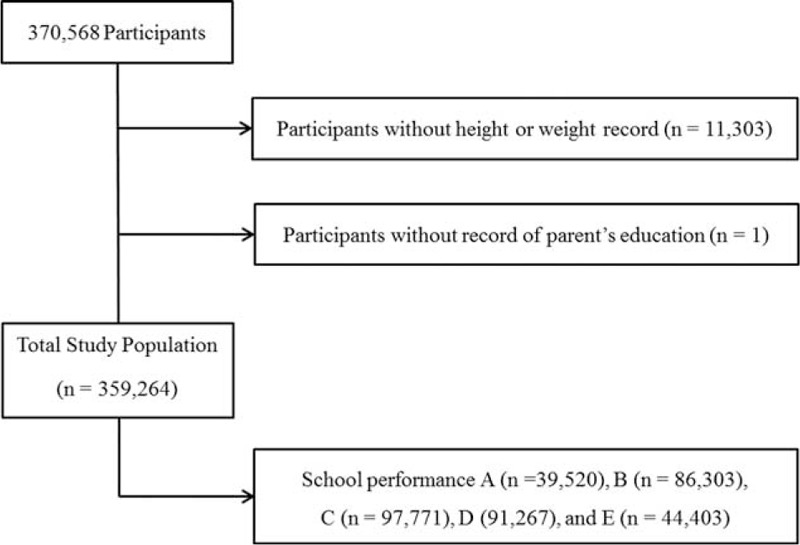
A schematic illustration of participant selection in this study. Of 370,568 participants, those with incomplete records were excluded. Data for a total of 359,264 participants from whom complete data were obtained were analyzed.

Of 370,568 participants, we excluded the following from the study: those who did not record their height or weight (11,303 participants) and those who did not provide the educational level of their mother (1 participant). A total of 359,264 participants (184,801 males and 174,463 females) from 12 to 18 years old were included in this study (Figure 1).

### Survey

Days of physical activity were measured as the days on which exercise lasted more than 60 minutes and was vigorous enough to raise the heart rate or respiration over the past 7 days.^16^ Obesity was categorized into 4 groups according to the Centers for Disease Control and Prevention guidelines for body mass index (kg/m^2^) for children and teens^17^: obese ≥95th percentile; overweight ≥85th and <95th percentile; healthy weight ≥5th and <85th percentile; and underweight <5th percentile. The regions of residence were classified into 3 groups by administrative district: large city, small city, and rural area. Participants were asked to classify their subjective self-assessment of health into 5 grades: very good, good, normal, not good, and very bad. Participants classified their stress level into 5 categories: I feel a lot of stress, I feel some stress, I feel a little stress, I rarely feel stress, and I do not feel stress. Participants were asked about the economic status of their family based on 5 levels ranked from the highest to the lowest. The parental education level was surveyed based on 4 categories: graduated college or over, graduated high school, graduated middle school or under, and unknown or no parent. The participants who do not know the educational level of their parents or who had no parents were not excluded because this could increase the missing values among relatively lower economic level participants. The regularity of meal time was surveyed based on the days that breakfast, lunch, and dinner were consumed over the past 7 days, as divided into 4 groups: 6 to 7 times a week; 3 to 5 times a week; 1 to 2 times a week; and 0 times a week. Drinking only a cup of juice or milk was not counted as having eaten breakfast, lunch, or dinner. Participants were also asked the frequency of eating fruits (not fruit juices), soft drinks, fast foods (such as pizza, hamburgers, or chicken), instant noodles, confections, vegetables, and milk over the last 7 days, and the responses were divided into 4 groups: ≥7 times a week; 3 to 6 times a week; 1 to 2 times a week; and 0 times a week. The participants were asked about their academic performance in their grade at school over the last 12 months. School performance was divided into 5 levels: A (highest), B (middle, high), C (middle), D (middle, low), and E (lowest).

### Statistical Analysis

The differences in general characteristics affecting performance at school were calculated using ANOVA for age and days of physical activity; Chi-square tests were used for sex, obesity, region of residence, subjective health, stress level, economic level, education level of father, education level of mother, regularity of breakfast, lunch, dinner and frequency of fruit, soft drink, fast food, instant noodle, confection, vegetable, and milk consumption (Tables 1 and 2).

**TABLE 1:**
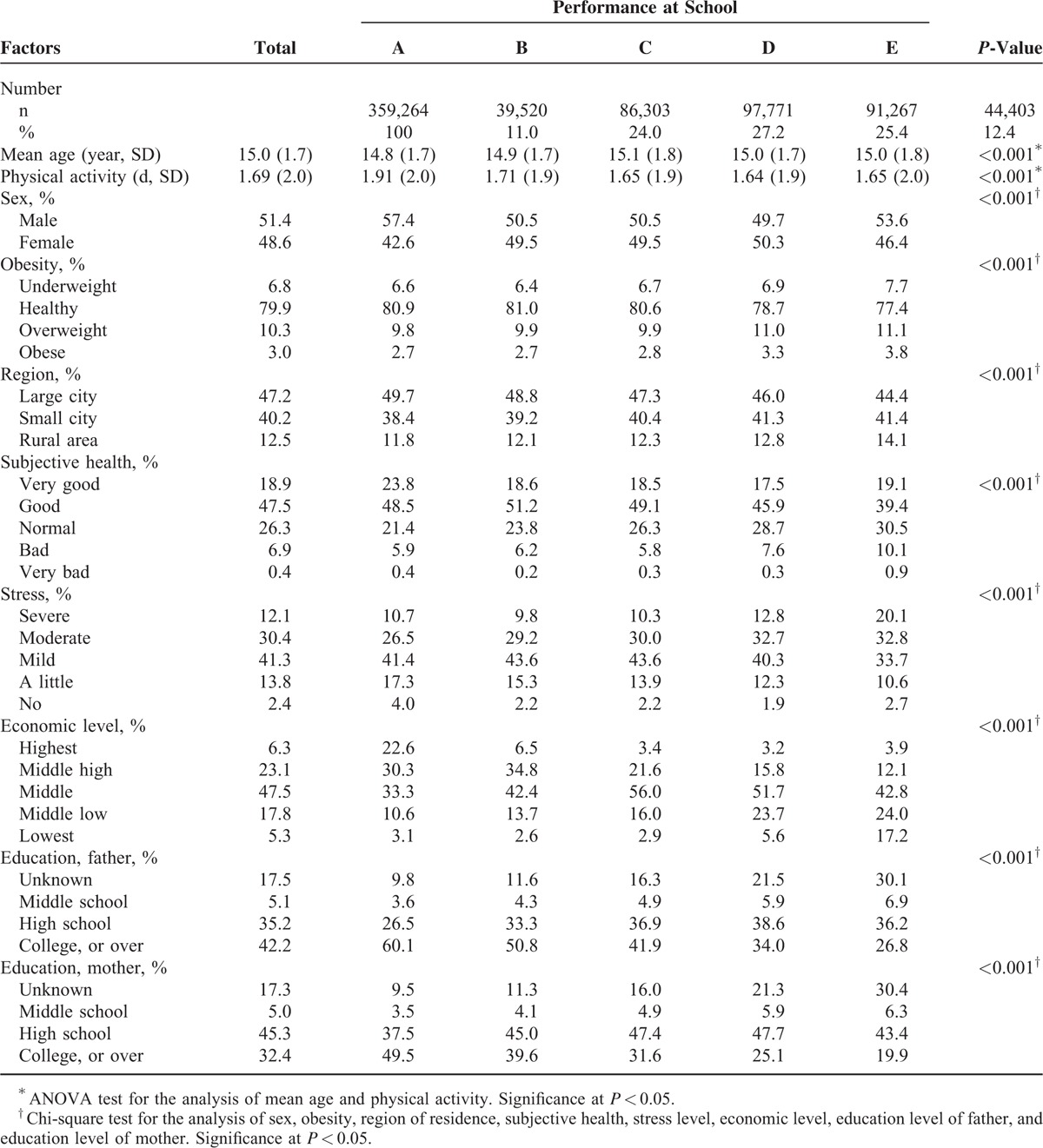
General Characteristics of Participants According to Performance at School

**TABLE 2:**
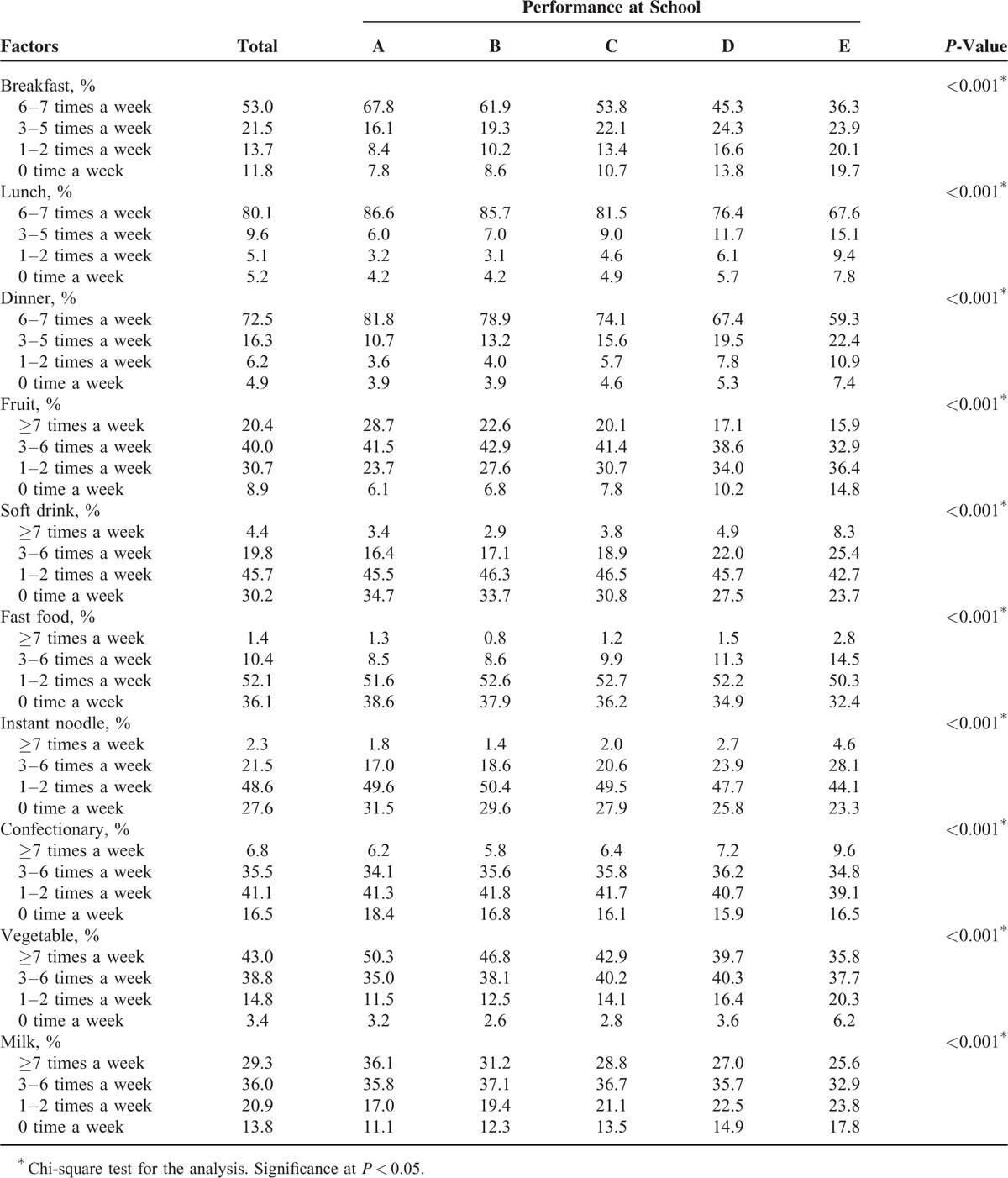
Diet Habit Rates of Participants According to Performance at School

Multinomial logistic regression with complex sampling adjusted for age, sex, obesity, region of residence, subjective health, stress level, economic level, education level of father, education level of mother, frequency of breakfast, lunch, dinner, and fruit, soft drink, fast food, instant noodle, confectionary, vegetable, and milk consumption (Table 3). Two-tailed tests were conducted and *P*-values lower than 0.05 were considered to indicate significance. Adjusted odd ratios (AORs) and 95% confidence intervals (CIs) were calculated. After applying the weighted values recommended by the KYRBWS, all results are presented as weighted values. The data were analyzed statistically using SPSS ver. 21.0 (IBM, Armonk, NY).

**TABLE 3:**
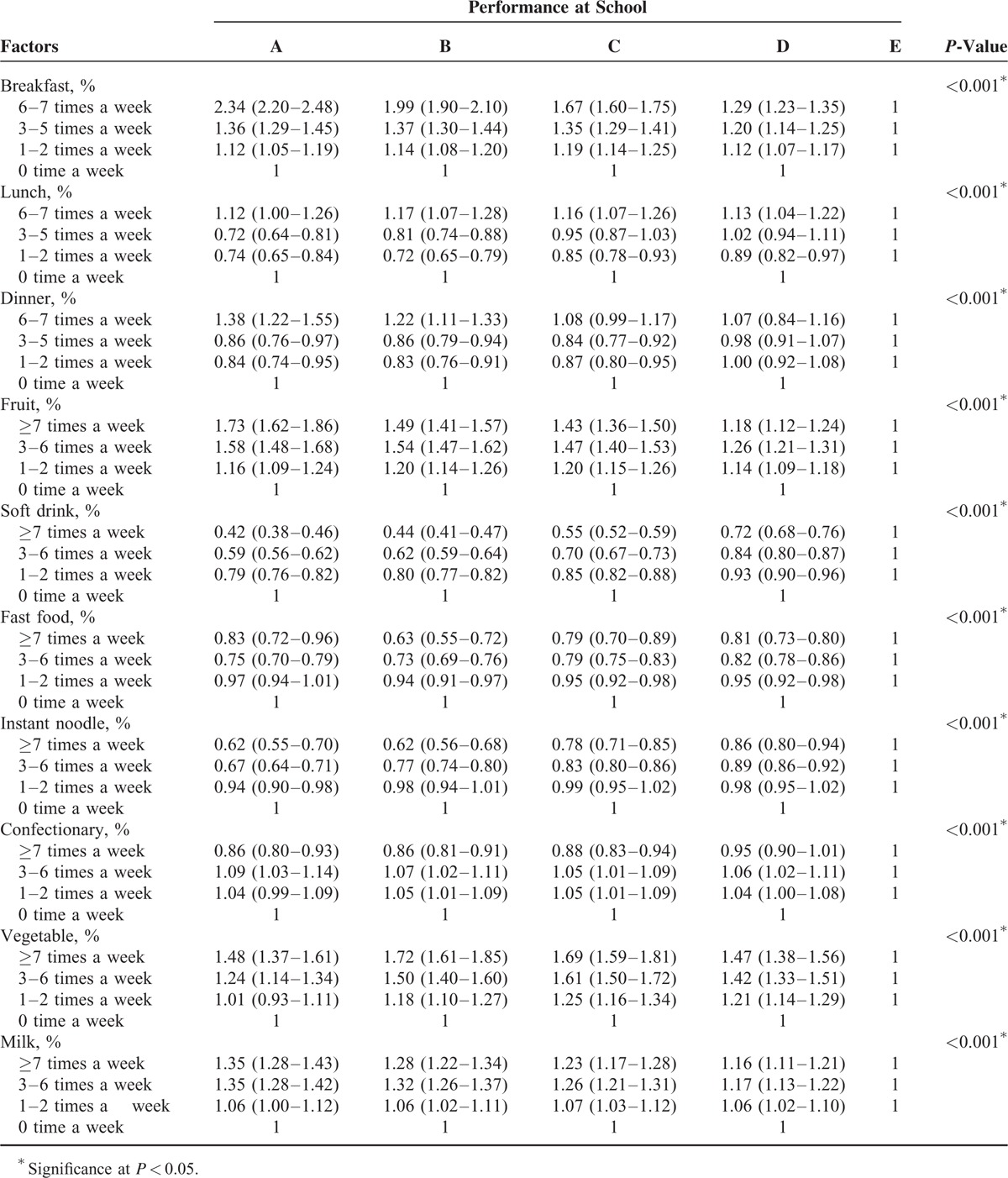
Adjusted Odd Ratios of Dietary Habit for School Performance Using Multinomial Logistic Regression Analysis With Complex Sampling

Structural equation modeling was used to explain the effects of diet factors on school performance while considering effects of other variables on both diet factors and school performance.^18^ Age, body mass index, subjective health status, and physical activity were defined as personal factors. Region of residence, economic status, and education level of parents were defined as SES. Frequency of meals and intake of various items were defined as the diet factors. Score was defined as school performance. Personal factors and SES were set as the exogenous variables, while diet factors and school performance were defined as the endogenous variables. Standardized regression weights were measured, and standardized total effects, direct effects, and indirect effects were calculated (Tables 4 and 5). The results were analyzed using SPSS Amos ver. 21.0.

**TABLE 4:**
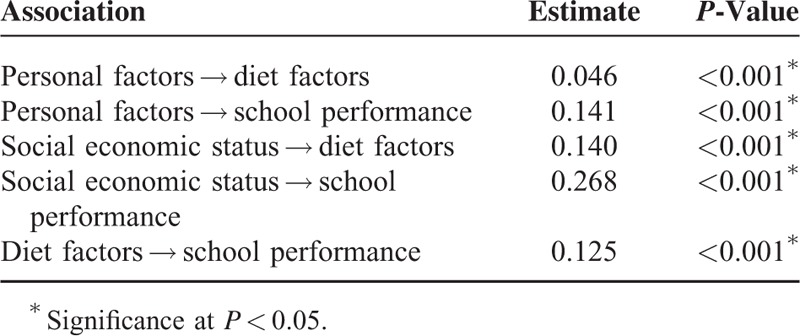
Standardized Regression Weight in Structural Equation Model

**TABLE 5:**
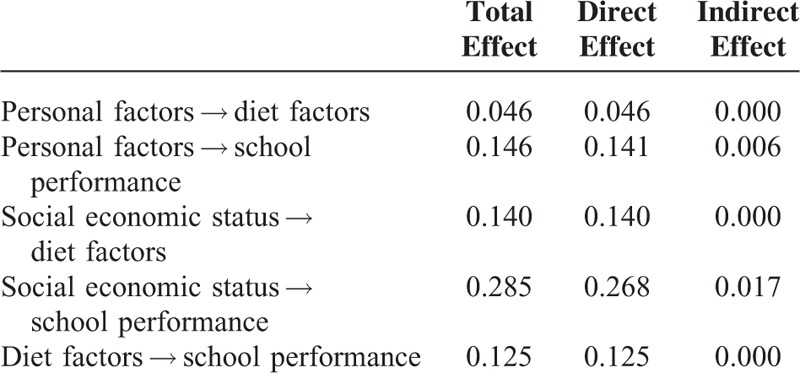
Standardized Total Effect, Direct Effect, and Indirect Effect in Structural Equation Model

## RESULTS

The demographic characteristics are summarized in Table 1. Reporting higher physical activity, being male, reporting a healthy weight, living in a large city, feeling subjectively healthy, feeling lower stress, being at a higher economic level, and having higher parental educational levels were associated with higher school performance (each with *P* < 0.001).

Diet habits are summarized in Table 2. The regularity of consuming breakfast, lunch, and dinner and frequency of fruit, vegetable, and milk intake were associated with higher school performance, while frequent intakes of soft drinks, fast foods, instant noodles, and confections were linked with poor school performance (each with *P* < 0.001). To evaluate possible associations among various dietary habits, we conducted a correlation analysis and confirmed that most of the Phi coefficients were very small (less than <0.3) (see Table S1, Supplemental Content, which illustrates the Phi correlations among eating behaviors).^17^

We performed a multinomial logistic regression analysis adjusting for confounding factors (Table 3). In group A, compared with never eating breakfast, eating breakfast frequently showed a high AOR with a dose–response relationship (1–2 times AOR = 1.12, 95% CI = 1.05–1.19; 3–5 times AOR = 1.36, 95% CI = 1.29–1.45; 6–7 times AOR = 2.34, 95% CI = 2.20–2.48, *P* < 0.001). Other school performance groups (B–D) also showed dose–response relationships eating breakfast (*P* < 0.001). In group A, having lunch and dinner 6 to 7 times a week showed high AORs of 1.12 (95% CI = 1.00–1.26) and 1.38 (95% CI = 1.22–1.55), respectively (each with *P* < 0.001). However, reporting less than 6 meal times a week showed a negative relation with school performance for both lunch (1–2 times AOR = 0.74, 95% CI = 0.65–0.84; 3–5 times AOR = 0.72, 95% CI = 0.64–0.81, *P* < 0.001) and dinner (1–2 times AOR = 0.84, 95% CI = 0.74–0.95; 3–5 times AOR = 0.86, 95% CI = 0.76–0.97, *P* < 0.001).

Compared with never eating fruit, eating more was associated with group A school performance with a dose–response relationship (1–2 times AOR = 1.16, 95% CI = 1.09–1.24; 3–6 times AOR = 1.58, 95% CI = 1.48–1.68; ≥7 times AOR = 1.73, 95% CI = 1.62–1.86, *P* < 0.001). Similarly, frequent milk consumption was related to group A performance with a dose–response relationship (1–2 times AOR = 1.06, 95% CI = 1.00–1.12; 3–6 times AOR = 1.35, 95% CI = 1.28–1.42; ≥7 times AOR = 1.35, 95% CI = 1.28–1.43, *P* < 0.001). Frequent eating of vegetables was also associated with school performance with a dose–response relationship (1–2 times AOR = 1.01, 95% CI = 0.93–1.11; 3–6 times AOR = 1.24, 95% CI = 1.14–1.34; ≥7 times AOR = 1.48, 95% CI = 1.37–1.61, *P* < 0.001). However, consuming more soft drinks (1–2 times AOR = 0.79, 95% CI = 0.76–0.82; 3–6 times AOR = 0.59, 95% CI = 0.56–0.62; ≥7 times AOR = 0.42, 95% CI = 0.38–0.46) and instant noodles (1–2 times AOR = 0.94, 95% CI = 0.90–0.98; 3–6 times AOR = 0.67, 95% CI = 0.64–0.71; ≥7 times AOR = 0.62, 95% CI = 0.55–0.70) was negatively associated with school performance with a dose–response relationship (each with *P* < 0.001). Frequent fast food consumption was also negatively linked with school performance (1–2 times AOR = 0.97, 95% CI = 0.94–1.01; 3–6 times AOR = 0.75, 95% CI = 0.70–0.79; ≥7 times AOR = 0.83, 95% CI = 0.72–0.96, *P* < 0.001). Although eating confections less than 7 times a week did not show an evident negative relation with school performance, eating them ≥7 times a week was negatively associated with school performance (≥7 times AOR = 0.86, 95% CI = 0.80–0.93, *P* < 0.001).

**FIGURE 2:**
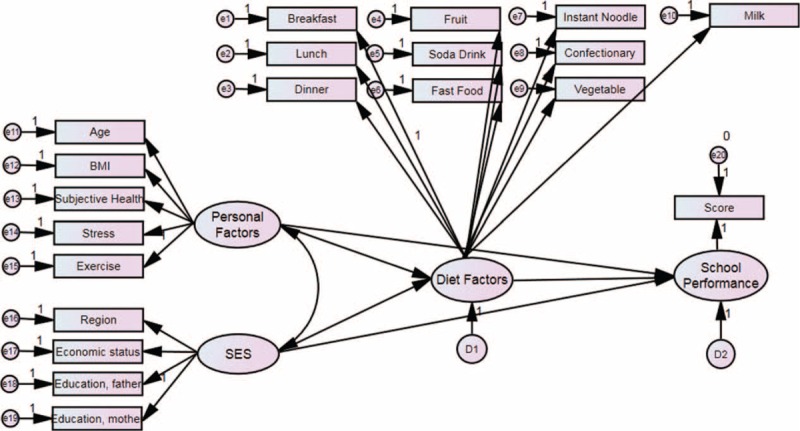
Structural equation modeling was used to explain the effects of diet factors on school performance while considering the effects of other variables on diet factors and school performance.

Standardized regression weights (direct effects) are calculated. The estimated values of personal factors to diet factor, personal factors to school performance, SES to diet factors, SES to school performance, and diet factors to school performance were 0.046, 0.141, 0.140, 0.268, and 0.125, respectively (each with *P* < 0.001) (Table 4) (Figure 2).

The standardized total effects were calculated using direct and indirect effects. The total effects of personal factors to diet factor, personal factors to school performance, SES to diet factors, SES to school performance, and diet factors to school performance were 0.046, 0.146, 0.140, 0.285, and 0.125, respectively (Table 5) (Figure 2).

## DISCUSSION

We found that the regular consumption of breakfast and frequent intake of fruits, vegetables, and milk contributed to high levels of school performance to varying degrees. Conversely, any frequency of soft drink, instant noodle, fast food intake, and eating confections ≥7 times a week negatively affected school performance. These relations between dietary habits and school performance were maintained after considering interactions with personal, socioeconomic, and dietary factors. To date, no study has comprehensively analyzed the dietary habits that are related to school performance after considering various factors. Moreover, this is the 1st study of the correlations between dietary intakes and school performance among Korean adolescents.

Of the participants, 11.8% consumed fast food more than 3 times a week in this study. This figure is comparable to that previously reported by the International Study of Asthma and Allergies in Children, which estimated that 13% of adolescents consumed fast food more than 3 times a week.^19^ As fast food generally contains poor nutrient content, as mentioned in the introduction, there are several concerns about the adverse outcomes of fast food. It is known that poor nutritional intakes that do not satisfy the recommended daily allowances for macro- and micronutrients are associated with significantly poorer attendance, punctuality, and grades at school, as well as with more behavior problems. These problems could be improved by adequate nutrition support in other studies of children.^2,20^ The poor nutritional composition of fast foods, which contain high amounts of fats and carbohydrates, may influence poor school performance. In animal and adult human studies, high fat and high carbohydrate diets are suggested to have detrimental effects on cognitive function, even after acute exposure over several days.^21–23^ Short-term ingestion of a high-fat diet (55% kcal from fat) impairs exercise capacity and cognitive function in both animal and adult human studies.^21,23^ Moreover, in animal studies, increased expression of genes related to inefficient fatty acid oxidation, such as uncoupling protein levels in mitochondria, are observed.^21^ These uncoupling proteins diminish metabolic efficiency (ATP production/O_2_ consumption), thereby impairing endurance performance.^21^ As fat cannot permeate the blood–brain barrier, substrate deprivation for energy production in the brain and insulin resistance are suggested as plausible mechanisms for the impaired cognitive function observed with high-fat diets.^24^ However, adults who consume high carbohydrate meals (54% kcal from carbohydrate) showed longer reaction times in cognitive performance tasks than those who consumed balanced meals, probably due to the increased availability of tryptophan to the serotonergic neurons involved in the sedative effect.^22^ A recent study experimentally proved that high saturated fat and refined carbohydrate diets induce impaired function in the frontal, limbic, and hippocampal systems, which perform learning, memory, and cognition functions.^25^ Several theories, including dietary-induced reductions in brain-derived neurotrophic factor (BDNF), oxidative stress, neuroinflammation, and an impaired blood–brain barrier, explain impaired brain function.^25^ Consistent with these theories, this study showed that fast food intake was related to poor school performance. Similarly, high carbohydrate foods, such as soft drinks and instant noodles, showed negative correlations with school performance in this study.

The relation between confections and school performance was inconsistent in this study. In 1 student study (9–22 years), it was reported that the intake of confections in the afternoon improved spatial memory, although there was contradictory results for attention.^26^ An afternoon snack may prevent starvation during daytime, which may impair brain function. However, confections are composed of refined carbohydrates or sugars,^27^ which were suggested to impair the frontal, limbic, and hippocampal systems, as well as their associated functions in learning, memory, and cognition if it is surfeited.^28^ Consuming confections more than 6 times a week was negatively related to school performance in this study (Table 3).

Fruits and vegetables were related to high levels of school performance. In children, a previous study demonstrated a significant correlation between executive cognitive function and snack foods but not with fruit or vegetable intakes, probably due to the small study sample and the limited number of variables considered, which might obscure the relations between fruit and vegetable consumption and executive cognitive function.^29^ However, several studies have demonstrated that high intakes of vegetables are related to good cognitive function in elderly populations.^30,31^ It was suggested that high fruit and vegetable intakes (4 or more portions/day; >350 g/day) are associated with statistically significantly increased level of antioxidants, such as carotenoids and alpha-tocopherol, whose blood levels were correlated with the results of cognitive function tests, such as the MMSE, Clock Drawing Test, and Dem Test.^32^ From the nutritional side, sufficient intakes of fruits and vegetables supply valuable micronutrients, such as vitamins C and E and minerals, required for brain metabolism.^33^ For instance, flavonoid intake dose-dependently reversed memory impairment by 40% to 70% in a mouse model.^34^ Moreover, because lutein and zeaxanthin are widely distributed and function in brain tissue and the macula of the retina, adequate intakes are crucial for both visual and cognitive functions throughout the lifespan.^35^

Milk was related to good school performance in this study. Dairy foods, including milk, were suggested to be beneficial to the neurocognitive functions of memory, vigilance, planning, and dichotic listening, probably due to better glucose tolerance in the brain and positive effects of bioactive peptides, colostrinin, proline-rich polypeptides, lactalbumin, vitamin B12, calcium, and probiotics.^36^

Our results showed positive relations between regular breakfast consumption and school performance in a dose-dependent manner. Several reports have suggested the beneficial effects of breakfast on cognitive performance and alertness.^9,37,38^ Regardless of supplement use, eating breakfast proved to be related to a smaller percentage of subjects not meeting two-thirds of the recommended daily allowance of valuable nutrients, including vitamin A (60.7% vs 43.0% for no breakfast vs breakfast, *P* < 0.001), vitamin C (35.6% vs 19.6% for no breakfast vs breakfast, *P* < 0.001), vitamin B-6 (35.6% vs 18.0% for no breakfast vs breakfast, *P* < 0.001), vitamin B-12 (20.7% vs 10.3% for no breakfast vs breakfast, *P* < 0.001), folate (23.7% vs 5.5% for no breakfast vs breakfast, *P* < 0.001), iron (34.8% vs 15.8% for no breakfast vs breakfast, *P* < 0.001), calcium (61.5% vs 38.8% for no breakfast vs breakfast, *P* < 0.001), phosphorus (36.3% vs 15.6% for no breakfast vs breakfast, *P* < 0.001), and magnesium (36.3% vs 21.4% for no breakfast vs breakfast, *P* < 0.001), which were difficult to compensate for through other meals in a European study.^39^ These results can be partially explained by the fact that eating regular breakfasts at home reduces the consumption of unhealthy snack foods during the day.^40^ In addition, eating breakfast may result in a more even distribution of energy and nutrient intake throughout the day. Therefore, it reduces obesity and energy shortages in the morning, which are negatively related to school performance in other studies of preschool children.^41^

In addition to these nutritional aspects, dietary habits themselves might influence school performance. Previous studies have demonstrated that overall diet quality, as indicated by the diet quality index, is independently related to academic performance, as subjects in 3rd (highest) diet quality index category were 30% less likely to fail a literacy assessment compared to 1st (lowest) diet quality index category (AOR = 0.70, 95% CI = 0.56–0.88).^13^ Dietary habits may reflect invisible factors, such as socioeconomic advantages and weight status, which can affect school performance.^13^ Health-related behaviors, such as eating breakfast, eating healthy foods, and avoiding junk foods, may be associated with good student compliance, which consequently improves school performance. It is possible that frequent fast food intake is correlated with undetected living circumstances, for instance, living without guidance or being of low SES.^42^ On the contrary, frequent breakfast intake implies that the participants live in conditions that allow and with parents who provide breakfast. It may also indicate that they are well-disciplined and self-controlled persons. It has been suggested that self-control is linked with the performance of desired behaviors and the inhibition of undesired behaviors.^43^ These circumstances may have significant effects on school performance by influencing school attendance, study duration, and personal characteristics such as steadiness and learning concentration. Moreover, it is possible that school performance affects dietary habits. For instance, students with good school performance might better knowledge of which dietary behaviors are good for health.

The present study, which adjusted for various factors including personal and socioeconomic factors using multinomial logistic regression analysis, is superior to previous studies. Furthermore, we adopted structural equation modeling to evaluate the relations among various personal, socioeconomic, diet factors, and school performance, thereby estimating the direct effects of diet factors on school performance (Figure 2). Diet factors influence school performance independently from the effects of other factors (direct effects) or depending on the influence of other factors (indirect effects). However, both diet factors and school performance are influenced by 3rd factors, such as personal and socioeconomic factors. For instance, socioeconomic factors are known to influence to school performance by mediating structural brain development.^44^ Therefore, it was possible that socioeconomic factors influence to diet factors as well as school performance and that there is no direct association between school performance and diet factors. To consider these issues, we analyzed the direct and indirect effects of each factor. Each factor demonstrated significant total and direct effects on school performance (Table 5). Personal factors showed a total effect of 0.146 and a direct effect of 0.141 on school performance. Similarly, social economic status showed a total effect of 0.285 and a direct effect of 0.268 on school performance. In comparison, the direct effect of diet factors on school performance was 0.125, which is considerable compared to those of other factors.

This study has several limitations. Although we tried to consider numerous confounding factors, including some personal and socioeconomic covariates such as region of residence, economic level, and parental education level, we cannot completely exclude the influence of these factors, such as parents’ occupations or family members living together. Moreover, as mentioned above, this study could not identify causal relations, such as possible reverse causality, due to its cross-sectional design.

As we mentioned in the method section, our classification system did not proportionally divide each level of school performance. However, this classification provided better information about the relations between the dietary habits and school performance than that obtained from a dichotomous classification. Another intrinsic limitation of this study is the accuracy of self-reported dietary consumption. As our survey investigated the frequencies of food intakes, the amounts of foods consumed could not be estimated. Moreover, because we retrieve the data only on the types of foods and there was no nutrient calculation, we were unable to quantify the nutrient exposure of the participants. However, our study population was representative of adolescents, and the survey was school based. Therefore, the reliability of the survey was predicted to be superior to those of the elderly or other general populations. Furthermore, we excluded uncompleted surveys, which might imply low confidence in the survey answers.

This study possesses considerable value due to its novel findings. This is a unique study based on a large, representative population in Korea. This study considered various kinds of foods to analyze the factors associated with school performance. Numerous covariates and their interactions are considered using standardized regression analyses, which enable us to minimize the confounding effects of other factors on the relations between certain foods or meals and school performance. Even after considering personal factors and SES, dietary habits eating fruit, soft drinks, fast foods, instant noodles, confections, vegetables, and milk consumption as well as regular consumption of breakfast, lunch, and dinner showed significant influences on school performance. Each dietary habit was independently related to school performance. Further study will be needed to elucidate the mechanisms involved in the relations between these dietary components and academic performance.

## CONCLUSION

The eating 3 times per day without skipping meals, especially breakfast, and frequent intakes of fresh fruits, vegetables, and milk were related to good school performance. However, consuming several processed foods such as soft drinks, instant noodles, fast foods, and eating confections more than 7 times a week showed correlations with poor school performance. This information about dietary habits has to be considered when we educate and consult on nutrition for adolescents.

## Supplementary Material

Supplemental Digital Content
