# Supplementary material for: Dietary Habits Are Associated With School Performance in Adolescents
Source: Medicine (Baltimore). 2016 Mar 25;95(12):e3096. doi: 10.1097/MD.0000000000003096 (PMC4998375; doi:10.1097/MD.0000000000003096)
Supplement: Supplemental Digital Content [file medi-95-e3096-s001.docx]

|  | Breakfast | Lunch | Dinner | Fruit | Soda | Fastfood | Noodle | Confectionary | Vegetable | Milk |
| --- | --- | --- | --- | --- | --- | --- | --- | --- | --- | --- |
| Breakfast |  | 0.416* | 0.423* | 0.154* | -0.116* | -0.070* | -0.088* | -0.009* | 0.111* | 0.103* |
| Lunch |  |  | 0.734* | 0.064* | -0.053* | -0.021* | -0.043* | 0.006* | 0.067* | 0.009* |
| Dinner |  |  |  | 0.076* | -0.042* | -0.016* | -0.024* | 0.008* | 0.073* | 0.026* |
| Fruit |  |  |  |  | 0.017* | 0.019* | -0.035* | 0.071* | 0.203* | 0.164* |
| Soda |  |  |  |  |  | 0.336* | 0.249* | 0.188* | -0.055* | 0.017* |
| Fastfood |  |  |  |  |  |  | 0.232* | 0.201* | -0.058* | -0.003* |
| Noodle |  |  |  |  |  |  |  | 0.157* | -0.069* | 0.019* |
| Confectionary |  |  |  |  |  |  |  |  | 0.005* | 0.027* |
| Vegetable |  |  |  |  |  |  |  |  |  | 0.133* |
| Milk |  |  |  |  |  |  |  |  |  |  |

**Supplemental content Table S1** The associations among eating behaviors

*Statistical significance, P <0.001
